# New extension software modules to enhance searching and display of transcriptome data in Tripal databases

**DOI:** 10.1093/database/bax052

**Published:** 2017-07-27

**Authors:** Ming Chen, Nathan Henry, Abdullah Almsaeed, Xiao Zhou, Jill Wegrzyn, Stephen Ficklin, Margaret Staton

**Affiliations:** 1 Department of Entomology and Plant Pathology, University of Tennessee, Knoxville, TN, USA; 2 Department of Genome Science and Technology, University of Tennessee, Knoxville, TN, USA; 3 Department of Ecology and Evolutionary Biology, University of Connecticut, Storrs, CT, USA and; 4 Department of Horticulture, Washington State University, Pullman, WA, USA

## Abstract

Tripal is an open source software package for developing biological databases with a focus on genetic and genomic data. It consists of a set of core modules that deliver essential functions for loading and displaying data records and associated attributes including organisms, sequence features and genetic markers. Beyond the core modules, community members are encouraged to contribute extension modules to build on the Tripal core and to customize Tripal for individual community needs. To expand the utility of the Tripal software system, particularly for RNASeq data, we developed two new extension modules. Tripal Elasticsearch enables fast, scalable searching of the entire content of a Tripal site as well as the construction of customized advanced searches of specific data types. We demonstrate the use of this module for searching assembled transcripts by functional annotation. A second module, Tripal Analysis Expression, houses and displays records from gene expression assays such as RNA sequencing. This includes biological source materials (biomaterials), gene expression values and protocols used to generate the data. In the case of an RNASeq experiment, this would reflect the individual organisms and tissues used to produce sequencing libraries, the normalized gene expression values derived from the RNASeq data analysis and a description of the software or code used to generate the expression values. The module will load data from common flat file formats including standard NCBI Biosample XML. Data loading, display options and other configurations can be controlled by authorized users in the Drupal administrative backend. Both modules are open source, include usage documentation, and can be found in the Tripal organization’s GitHub repository.

**Database URL: Tripal Elasticsearch module:** https://github.com/tripal/tripal_elasticsearch

**Tripal Analysis Expression module:** https://github.com/tripal/tripal_analysis_expression

## Introduction

Tripal is an open source toolkit for construction of online genome databases (1, 2). Tripal is built on the Drupal content management system (www.drupal.org) and stores data in the recommended standardized biological database schema, Chado (3). Both Tripal and Chado are members of the Generic Model Organism Database (GMOD) collection of open source and interoperable software tools (http://gmod.org). Tripal consists of a core set of modules that encompass a variety of common biological and genetic data types, such as organisms, sequence features and genetic markers which include attributes such as genus and species, nucleotide residues and marker locations, respectively. The software has been deployed for numerous genetic and biological data websites, with over 15 active sites listed on the Tripal information web page (http://tripal.info/sites_using_tripal). The adoption of this standardized web database software platform has numerous advantages, including leveraging code developed across different groups, standardization of data storage formats and an active mailing list and developer community for support.

The Tripal community encourages individual developers to extend and customize the software and provides helpful tools for this, including an application programming interface (API) and flexible data display templates. By leveraging these features, user-contributed extension modules provide additional functionality, including the upload and display of new data types such as transcriptome assemblies by Tripal Analysis Unigene (https://github.com/tripal/tripal_analysis_unigene), BLAST sequence similarity results by Tripal Analysis BLAST (https://github.com/tripal/tripal_analysis_blast) and gene ontology annotations through Tripal Analysis GO (https://github.com/tripal/tripal_analysis_go). The Tripal.info website provides a system for developers to register their extension modules, with descriptions of functionality, development status (from in-development to ready-for-use), compatible versions of the Tripal core and compatible versions of the Chado (http://tripal.info/extensions). Developers are also able to join the Tripal organization on GitHub (https://github.com/tripal) and contribute their modules in the centralized Tripal repository, thus encouraging discoverability, collaboration and communication among developer groups.

Despite the rich functionality already available, including storage of gene or assembled transcript sequences and their functional annotation, Tripal does not yet have robust search, storage or display of expression information or the biological samples queried in expression studies. As next generation sequencing instruments with high-throughput and low cost became available in 2005 and widely adopted by 2009, RNA sequencing (RNAseq) has become the most common method for assaying gene expression (4–6). This is reflected by the rising percentage of RNA records in public databases. In February of 2017 the NCBI sequence read archive (SRA) contains 518 625 records for RNA sequencing runs from Eukaryotes, representing 46% of the total Eukaryote sequence records.

A gene expression experiment seeks to quantify changes in transcript abundance across different samples; this may take the form of a broad survey (gene atlas) across many tissues, time points or developmental stages, or as a targeted profiling of changes induced by an experimental treatment, often abiotic or biotic stress (7–9). To increase the reusability of data and the comparison of data across different experiments, community databases need to capture as much metadata as possible about these biomaterials (10, 11). Once RNA is extracted and sequencing performed, a standard bioinformatics analysis incorporates sequence quality control steps such as adapter removal, *de novo* assembly if a reference genome is not available, mapping of reads to the reference assembly or genome, quantification of reads per gene or transcript and normalization of data (12, 13). These normalized values are comparable across biomaterials and often visualized as a heatmap for interpretation. Numerous software tools are available for each step of the analysis, and the choice of tool software, version and user-specified parameters influences final results (14, 15). Preserving methodological details is critical for appropriate data interpretation and to enable reuse, reproducibility and comparison among datasets.

For a community genome database, users can mine gene expression data to identify candidate genes or transcripts for further study based on information about expression patterns across different tissues and conditions. To further this goal, we have developed two new publicly available Tripal extension modules. The Elasticsearch module enables more efficient and flexible searching powered by the Elasticsearch software (https://www.elastic.co). With a broad site-wide search, users can enter any keyword, from a software tool name to a transcript name to a metabolic function, to find results across the entire Tripal site. Further, database developers may build customized search interfaces for specific data types with advanced filtering options. A second Tripal module, the Tripal Analysis Expression module, has been developed to store and display gene expression levels across multiple tissues and conditions derived from sequencing transcriptome data. For each sequencing sample, a biomaterial record is created and all attributes such as individual, tissue and experimental condition are added as ontology-tagged, searchable fields. Normalized expression values for each transcript or gene from individual biomaterial are stored. The resulting user interface of a bar chart or heatmap provides an intuitive visual representation of how genes or transcripts are expressed in an organism, providing important clues about biological function. Together, the modules provide significant additional functionality for users to explore RNASeq data, transcriptome assemblies and transcript expression levels.

The code for both modules is available via GitHub. They are currently in use at the Hardwood Genomics Web (http://hardwoodgenomics.org), a database that houses genetic and genomic data from hardwood tree species. Both modules are build in PHP and work with Drupal 7.x as an extension to Tripal version 2.x. Linux is the recommended operating system for Drupal installations. The modules currently do not support Drupal version (8), as the core Tripal modules have not yet transitioned, however, Drupal 7 is in long term support mode by the Drupal community. The modules are compatible with Chado versions from 1.2 to 1.31. Source code and developer guides are open source and made available through GitHub under the GNU General Public License 3. We welcome developer and user feedback, including bug reports and questions, through the GitHub issues queue.

## Tripal Elasticsearch module

### Overview

The Drupal content management system, which forms the base for all Tripal sites, provides its own built-in searching system. However, this system suffers from several disadvantages that render it either slow or unusable for some Tripal databases. First, the Drupal search system only indexes the HTML-rendered text content for Drupal nodes; in Drupal, nodes are individual pieces of content rendered as a web page. This is a major impediment to intelligent searching of a Tripal site, where the biological data that is stored in the Chado database schema is ignored unless it is part of a node. Even when data is displayed as part of a node, the biological context of the data may be lost. The native Drupal search also does not offer fuzzy or partial word matching. Finally, the indexing procedure for searching is very inefficient for sites with hundreds of thousands of records, leading to very slow indexing and searching for Tripal sites dealing with very large numbers of records.

**Figure 1.**
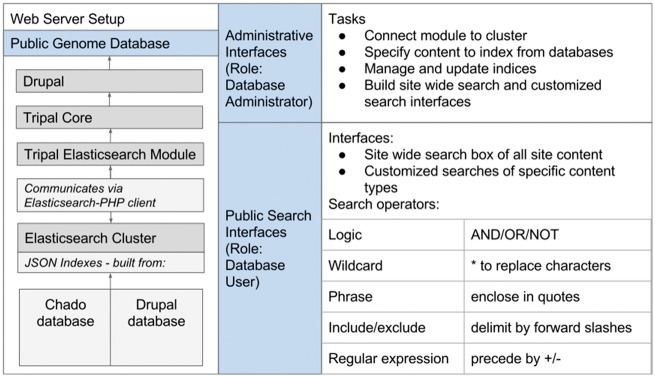
The Tripal Elasticsearch module depends on a number of other software packages. It works within the Drupal content management system and requires the core Tripal modules. To build indexes and run efficient searchs, it relies on an Elasticsearch cluster, which it communicates with via the Elasticsearch-PHP client. After installation of the software, all functionality can be controlled from web interfaces. The Drupal administrative user can configure and set up site-wide and customized search interfaces. Once those are active, all website users can perform fast, efficient searching with flexible search operators.

In order to provide site-wide searching functionality and flexible, efficient access to the data hosted in Chado, we developed the Tripal Elasticsearch module to integrate the Elasticsearch engine with Tripal sites. The Tripal Elasticsearch module leverages this search engine to provide both generic site-wide searching and the ability to build customized search interfaces for specific Chado database tables (Figure 1). Written in Java, Elasticsearch is open source software that provides a powerful, fast and feature-rich search engine. Elasticsearch clients are available in a variety languages, including PHP, the language of Drupal and Tripal (https://github.com/elastic/elasticsearch-php). Elasticsearch is designed to be used in distributed environments and thus is highly scalable, a critical concern with the quickly growing amount of available genomic data for many organisms. Elasticsearch indexes data in schema-free JSON documents that are stored on the web server and enable very fast look-up of search terms. The index files can be split into pieces, called shards, across many servers and support is provided to search and index multiple source databases. The Tripal Elasticsearch module opts to use the built-in query_string query method provided by Elasticsearch, which provides common search methods such as AND/OR/NOT operators, wildcard (*) search, phrase search with quotes, and  ± (include/exclude) operators.

The Tripal Elasticsearch module utilizes the Elasticsearch-PHP client to interact with the Elasticsearch cluster. For site-wide indexing, the full Hyper Text Markup Language (HTML) contents of all pages on the site are indexed. This is accomplished by first querying the Drupal database for all node IDs, as all individiual web pages are stored as nodes in Drupal 7. The module uses these IDs to query the site and extract the HTML contents for each page. The HTML strings are processed and, using the Elasticsearch-PHP client, turned into Elasticsearch indexes. For table-specific indexing, the process is slightly different. Instead of querying HTML, the module extracts data content from the specific Chado database tables and fields specified by the administrative user, and then uses the Elasticsearch-PHP client to create an Elasticsearch index specific to this dataset. A customized form and content search block are automatically created for this index, with the administator able to customize the form fields, the placement of the content block on the website, and the URLs to link the results to appropriate pages.

### Administration interface

**Figure 2.**
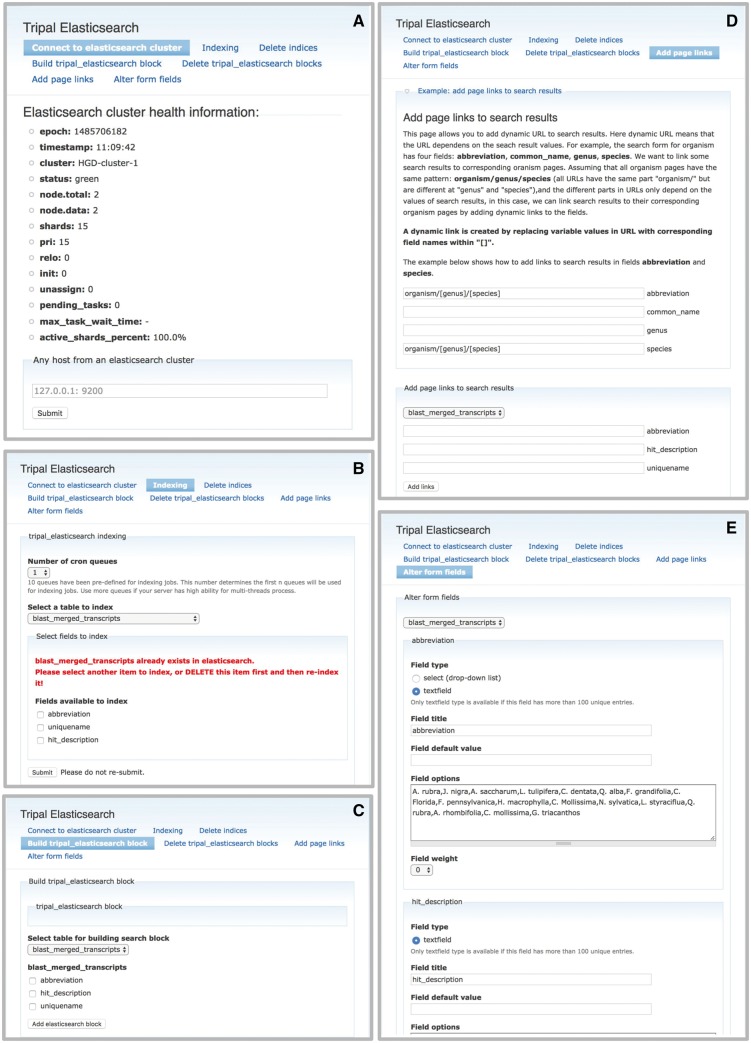
The Tripal Elasticsearch module administrative pages provide centralized control of data indexing and construction of search interfaces. (**A**) The module first needs to be connected to a running Elasticsearch cluster. (**B**) The user may opt to index the entire site by selecting ‘index_website’ or select individual tables and fields from the database. The index jobs are automatically launched via cron. (**C**) Based on available search indexes, a search form can be built and placed in a Drupal content block for display to users. (**D**) For each search block, the search results are linked to site content by constructing custom URLs (universal resource locator). The administrative page gives an example of how to use search results as tokens in the construction of URLs. (**E**) The search form can be customized for the best final user experience.

The Elasticsearch software can be installed on the same server as the Tripal site or on a remote host or cloud host. After this installation, the remaining setup can be controlled entirely through the graphical user interface (GUI) administrative backend provided by the Tripal Elasticsearch module. The module can be downloaded and enabled through Drupal in the same way as other Tripal extension modules. Next, the site administrator can connect the module to the local or remote Elasticsearch cluster through an administrative page. After a successful connection, health information of the cluster will be displayed (Figure 2A).

Next, site administrators can select the type of database content they would like to index and create cron jobs that will create those indexes. Administrators can also delete the indexes. One option is to create a site-wide index, where the module extracts the Hyper Text Markup Language (HTML) source code of all published Drupal nodes, excluding sequence strings, and then indexes these html strings using the Elasticsearch engine. By doing this, all published web pages become indexed and searchable, regardless of content type or location of the underlying data in the database. The module also implements Drupal insert, delete and update function hooks so that any modification to site content will be detected by the module and corresponding modifications to the Elasticsearch indexes will be made. The required modifications - adding, removing or altering individual page indexes - are submitted as jobs to the cron queue.

Alternatively to the site-wide search, the administrative page pre-populates a drop-down menu box with all tables from both the Drupal and Chado database schemas, allowing the administrator to select one for individual indexing (Figure 2B). The module will automatically detect which tables have been previously indexed and warn the user to prevent multiple indexes of the same data. To further refine administrator control of indexing, individual fields from a table may be included or excluded from the index. This individual table indexing is useful to design advanced search forms for subsets of data in the database or to incorporate searching of content in Chado that is not displayed in Drupal nodes.

Both site-wide and individual table indexing can be time consuming for large datasets, so the Tripal Elasticsearch module enables concurrent indexing jobs. By leveraging the cron queue functionality in Drupal (https://api.drupal.org/api/drupal/modules!system!system.api.php/function/hook_cron_queue_info/7.x), administrative users can opt to use up to ten simultaneous queues for the indexing process. Tripal Elasticsearch module also requires the Drupal module Queue UI, which can be used to monitor and manage cron queues (https://www.drupal.org/project/queue_ui). Indexing jobs are then divided and assigned evenly across the specified number of cron queues, and the queues are managed independently and concurrently by the cron daemon. Using more queues can largely decrease the time of indexing, but it also uses more memory and processors. The number of queues should be selected judiciously based on available server resources. One successful strategy to prevent interference with the responsiveness of a live production site is to run the indexing on a development server, then move the Elasticsearch index files to the primary web server.

After a table is selected and indexed, the administrator can build a search block to display to site users (Figure 2C). A Drupal block is a flexible unit of content that can be configured to display on all site pages or only on certain pages, and it can be placed in specific page locations such as a sidebar or header. Next, the administrator must enter information about how individual search result columns should link to content (Figure 2D). The search results themselves are available as tokens for building a URL to appropriately link each search result. For example, a gene search may return a table with the gene name and its organism. Each of these fields may be linked to the most appropriate place; i.e. the gene name to its feature page and the organism name to the organism page. Once the block and results links are built, the final step is to customize the search form if needed. The administrative user can select which of the indexed fields to expose as search boxes, the user input type (enter free text or select options from a dropdown), the field label, and the order of the fields in the display (Figure 2E).

### User interface

**Figure 3.**
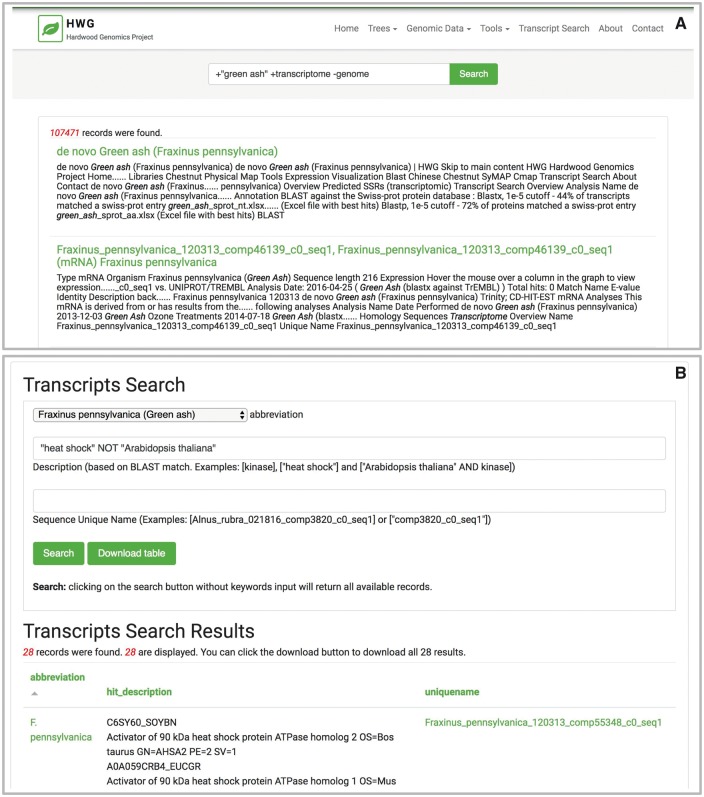
The Tripal Elasticsearch module builds content blocks for search forms. Using the Drupal block system, a search block may be displayed on every page, on its own dedicated page or on a specific page or pages across the site. Screenshots are taken from the Hardwood Genomics Web where the Tripal Elasticsearch module is in production use. (**A**) The generic site-wide search block includes a single, global search field and search button. **(B**) A table-specific search form provides additional search fields for more advanced filtering of results. Here, transcript results are quickly returned from a database containing over 500 000 transcript records and >13 600 000 BLAST hits.

The Tripal Elasticsearch module provides a simple input box for the generic site-wide search (Figure 3A) and the administrator-customized form for a table specific search (Figure 3B). While the Elasticsearch software provides over a hundred searching methods, the Tripal Elasticsearch module uses a comprehensive search method called *query_string* as its default search method. With this search method, users can build advanced queries, for example by enclosing exact search phrases with quotes, by prefixing words with ‘+’ or ‘-’ to require or exclude from results, and by using *AND*, *OR* and *NOT* operators. For better performance only the first 100 search results are returned by the generic site-wide search by default, but this can be altered by the site administrator. Search results are displayed with a web page title link and a portion of web page content with keywords in bold and italics, to provide context for the matching keyword. For a table-specific data search, 1000 search results are returned by default. Search results are displayed in a paginated table and can be sorted by clicking the table headers. A download button is provided to download all search results as a comma-separated value (csv) file.

Like the search forms which are embedded in a Drupal block, search results from individual tables are also displayed in independent Drupal blocks. This makes it possible for the Tripal Elasticsearch module to display aggregated data, and to functional as a faster alternative to Drupal Views, a widely-used core Drupal module. Administrative users can build a custom data view by using the same procedure for building a custom search block for desired data and then disabling the search form blocks. The populated result block will remain and may be display on any page in the site.

### Example of customized transcript search in HGD using materialized views

As a highly normalized schema, Chado often stores related data across many different tables. To speed page rendering, administrators often use materialized views. Materialized views are tables built to hold the results of a common database query, often a query that joins multiple tables or filters data. By storing the query results in a materialized view, the results do not have to be rebuilt for new page loads and can be quickly returned. The Tripal core includes a module for administrators to create, populate and update materialized views. The Tripal Elasticsearch module only indexes and creates a search block from a single table, so a materialized view is useful for building an advanced search interface that searches content normally distributed across multiple tables. For example, a site with RNASeq data may provide a search form that incorporates searching by transcript unique name, transcript functional annotation and organism. In this example (Figure 3B), the materialized view is created to aggregate data from three database tables: *chado.organism*, *chado.blast_hit_data* and *chado.feature*. The Tripal Elasticsearch module can be used to index the new materialized view and build a search form with the requisite custom fields. This transcript search example could easily be extended to include other types of functional annotation, such as KEGG results, gene ontology terms, phenotype associations and more. Once the custom materialized view is indexed by the module, it can be deleted from the database to reduce redundancy and minimize filesystem storage space.

## Tripal Analysis Expression module

### Overview of functionality

**Figure 4.**
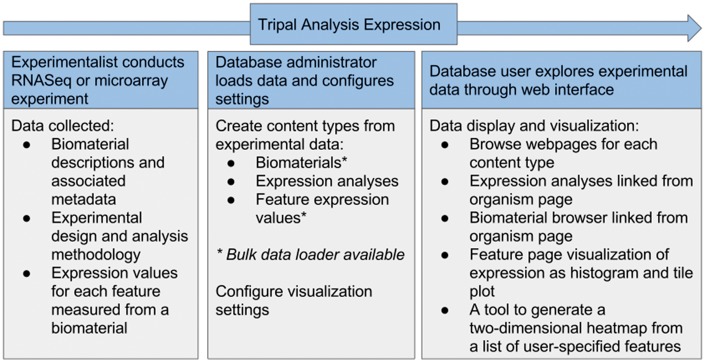
Flow of data in the Tripal Analysis Expression module. Either RNASeq or microarray experimental data can be utilized and needs to include biomaterias, design and analysis methodology and expression values. From within a web interface, a site administrator can add and configure this content, yielding a number of visual interfaces for users to explore the data.

**Figure 5.**
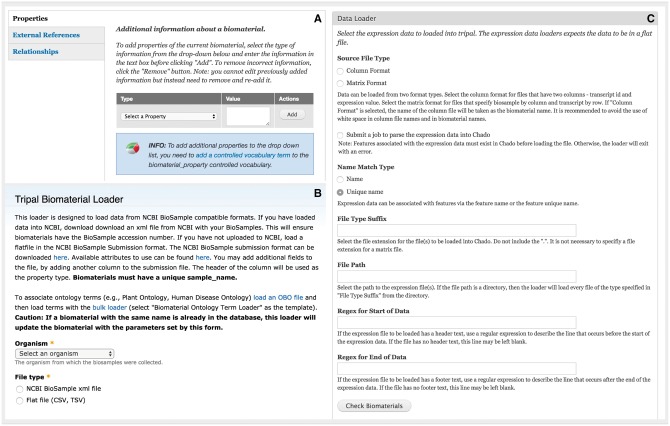
Administration interface of the Expression module. (**A**) Administrators can add customizable property fields to biomaterial record to provide metadata about experimental conditions, tissue, developmental stage, etc. These fields must be registered as a part of a controlled vocabulary through the core Tripal Controlled Vocabulary module. (**B**) The module is able to bulk load biomaterials from a CSV file or an NCBI BioSample XML formatted file. (**C**) The module can load expression data in column or matrix formats. These formats are described at the top of the page. The interface also allows the administrative user to specify regular expressions to exclude any header or footer text in the file that should be ignored such as column headings.

Tripal has existing support for aspects of RNA sequencing experiments, including assembled transcripts and associated *in silico* functional annotation from BLAST (16), InterProScan (17) and KEGG (18) analysis. The Tripal Analysis Expression module expands this support by enabling storage and display of a gene expression experiment, including a description of the experiment, the biological samples (biomaterials) and the normalized gene expression values (Table 1). The module is able to accept either RNASeq or microarray expression assays (Figure 4). To build a gene expression analysis record, first an administrator needs to add biomaterial records. At a minimum, the biomaterial record must hold a name, a description, and a provider contact, and it must be linked to an organism. However, this content type is flexible and expandable in order to store the many different possible types of metadata that may be associated with a biomaterial. Customizable property fields associated with a controlled vocabulary may also be added to fully describe a record (Figure 5A). This could include information such as tissue, treatment, life stage, or geographic location. The list of properties may be expanded to suit the needs of each experiment by importing new controlled vocabularies or adding new custom controlled vocabulary terms. Both options are available through the Tripal core. The module also provides for database cross referencing, where records link out to other databases such as NCBI’s Biosample database (19). The biomaterial content type may be used in a Tripal site independently without adding information about an expression experiment. It could be useful for describing biomaterials for other related-omics experiments such as genotyping, DNA sequencing, proteomics or metabolomics.

**Table 1.** Many different types of data may be produced related to an RNA sequencing experiment, and in many cases, there already exists a supporting Tripal module that accepts standard data file formats and encourages use of appropriate controlled vocabularies

Type of data	Tripal module	Upload file format	Controlled vocabulary	Corresponding NCBI database
RNA Sequence read	Feature (core)	Fasta	Sequence ontology	Sequence read archive
Assembled transcript sequence	Feature (core) and analysis unigene (extension)	Fasta	Sequence ontology	Transcriptome shotgun assembly
Gene sequence	Feature (core)	Fasta	Sequence ontology	Gene
BLAST result	BLAST (extension)	XML	Gene ontology	NA
KEGG result	KEGG (extension)	Tab-delimited	Gene ontology	NA
Biomaterial	Analysis expression (extension)	XML or tab-delimited	Species-dependent^a^	BioSample
Gene expression values	Analysis expression (extension)	Matrix or column files	NA	Gene expression omnibuss
Gene expression experiment methods	Analysis expression (extension)	Descriptive text	NA	BioProject

Data may be sourced from or uploaded to a corresponding database in NCBI.

a Biomaterials may be associated with multiple controlled vocabulaties, often species-specific. For example, plant samples may be described by anatomical structure and development stage with the Plant Ontology (20), by phenotype with the Plant Trait Ontology or by stress treatment with the Plant Stress Ontology. All of these ontologies are available through Planteome (http://planteome.org/).

After biomaterials are established, a full gene expression experiment may be described, starting by creating a new expression analysis record. The Tripal core already provides a generic analysis content type to represents a bioinformatic analysis of a dataset. An analysis record is useful for linking all data in the database back to the relevant materials and methods used to generate the data. To customize this for a gene expression experiment, the Tripal Analysis Expression module extends the analysis content type and adds fields for laboratory and data analysis protocols. If a microarray was used, there is an option to specify the array platform and design. It also enables the upload of the normalized gene expression data associated with the gene expression analysis record. In the database, this links each expression value to an individual feature (a gene, a transcript or other sequence) and to a biomaterial.

### Administration interface

After installing the Tripal Analysis Expression module, administrators or curators can utilize web forms to create new biomaterials and expression analysis content. For large sets of data, bulk import is also available. For biomaterials the provided data loader can parse BioSample records downloaded from NCBI in XML format (19) or derive data from a tab-delimited text file (Figure 5B). The data is stored into the Chado biomaterial tables (biomaterial, biomaterial_dbxref, biomaterialprop, etc.). Another data loader provides the ability to enter expression values into the Chado schema from common expression data formats (Figure 5C). The first option is column format, where a folder with individual files is provided with each file corresponding to a biomaterial and containing one gene per line, followed by a space or tab and the numerical expression value. A second option is to provide a large tab-separated value file with gene names beginning each line and columns of values corresponding to biomaterials. Examples of both file formats are provided within the module documentation. The module assumes that expression values are normalized prior to entry and no statistical manipulation of the data is performed. In an effort to maintain compatibility with current and past databases with the Chado schema, the Tripal Analysis Expression module does not create any new database tables. Instead the module utilizes existing Chado tables from the MAGE (microarray gene expression), organism, contact, sequence and companalysis table groups (3).

The Tripal Analysis Expression module provides full administrative functionality for each content type. The site administrator can sync, delete, and alter settings for each content type provided by the module. The Tripal Analysis Expression module is completely separate from the Tripal Elasticsearch module; some sites may choose to use one but not the other. To account for this possibility, standard Drupal views-based searches are automatically provided for biomaterials and gene expression analysis records.

### User interface

Users can access biomaterials associated with an organism directly from the organism page in a Tripal site. The Tripal Analysis Expression module interacts with the organism module using the Drupal hook system and uses the standard Tripal page template to add a link to biomaterials on the organism page sidebar. When a user clicks on this side menu link, a list of biomaterials is displayed, with multiple pages if the list is longer than ten. Clicking through to an individual biomaterial provides information including all associated metadata properties and external database references. All page templates are fully customizable and site developers may override defaults to alter menu items, link locations, paging options, etc. Further, if a site administrator wishes to create a page dedicated to exploring all biomaterial records outside of the organism page, a dedicated page may be created with Drupal Views or Tripal Elasticsearch.

**Figure 6.**
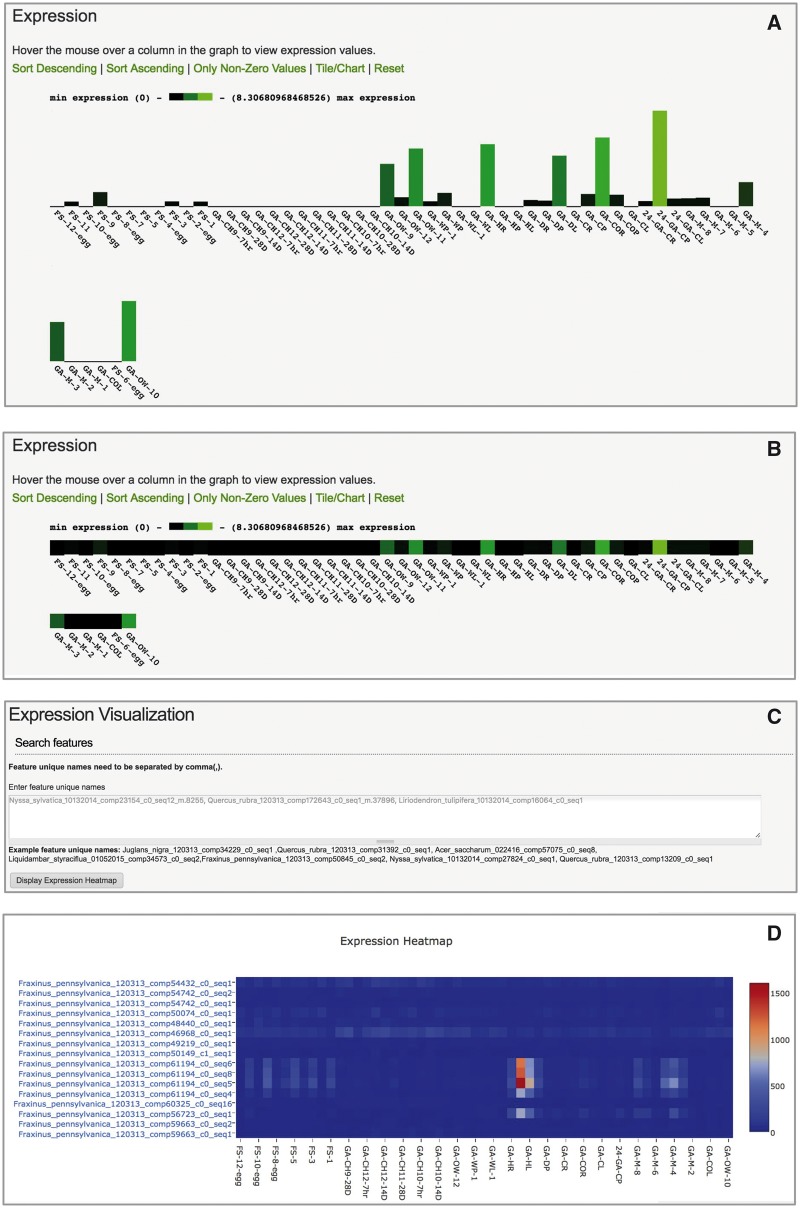
User interface of Tripal Analysis Expression module. (**A**) The feature page initially displays expression values as a bar chart. (**B**) Users may toggle the the barchart to a one-dimensional heatmap. (**C**) An interactive tool allows users to paste in a list of feature ids of interest. (**D**) The tool will create a two dimensional heatmap of the feature expression values across all available biomaterials. This heatmap displays ubiquitin transcripts (top eight rows) vs. heat-shock transcripts (bottom eight rows) from Fraxinus pennsylvanica (green ash). Hovering over the boxes with red and orange color in the online heatmap reveals that they are derived from petioles and leaves exposed to heat stress (40 °C for 24 h).

To explore gene expression values, the Tripal Analysis Expression module also hooks into the Tripal Feature module. For each feature with expression data, a link labeled ‘Expression’ is added to the side menu on the feature page. Clicking on this link generates a one dimensional heatmap figure using the JavaScript D3 library (https://d3js.org/). The user has the option of altering the heatmap figure by removing biomaterial libraries that do not express the feature or sorting the biomaterials by expression value. The expression figure may be displayed as a bar chart (Figure 6A) or as a one-dimensional heat map (Figure 6B). The biomaterial names displayed on the x-axis are usually short identifiers, so if the user hovers their mouse pointer over a biomaterial, a pop-up display with the full biomaterial description appears.

The module also provides a tool for users to enter a list of feature names (Figure 6C) and display their expression data as a two-dimensional heatmap (Figure 6D). This tool comes embedded in a Drupal content block that may be placed on any page or embedded in its own page. Users can directly enter a list of feature unique names or IDs of interest, or obtain features via transcript or feature search interfaces that are built by applying the Tripal Elasticsearch module. The heatmap is created with the Plotly javascript graphing library (https://plot.ly/javascript/) which provides interactive tools enabling the user to pan, zoom and download the image inside the browser window. Users may perform further image exploration by selecting the ‘save and edit plot in cloud’, which loads their current image and its underlying data into the public Plotly cloud-based workspace tool (https://plot.ly/create/) with many additional data visualization and filtering options.

### Example of exploring differential expression with the Tripal Elasticsearch and Tripal Analysis Expression modules

Both modules are currently installed on the Hardwood Genomics Web, a site housing forest tree transcriptome data. Using this database we can illustrate how the modules can be used together to explore the expression patterns of a group of transcripts. For example, a user may want to explore expression patterns in transcripts originating from heat shock gene family members in *Fraxinus pennsylvanica* (green ash). This species has a published transcriptome with 107 611 transcripts derived from *de novo* assembly of RNA sequence data from 55 biomaterials, including several stress treatments such as heat, cold, drought, mechanical wounding and varying levels of ozone concentration (21). Heat shock proteins were initially discovered as an induced response to heat stress in Drosophila (22) and plant homologs have also been observed increasing in expression under heat stress as well as other types of stress including cold and wounding (23, 24). To explore if heat shock proteins are induced across the various stressed biomaterials in *F. pennsylvanica*, a user might first use the transcript search interface with the phrase ‘heat shock’ in the BLAST match description (Figure 3B). The organism may be filtered by selecting ‘*Fraxinus pennsylvanica*’. As of May 2017, this yields 302 putative heat shock transcripts. For comparison purposes, reference transcripts likely to be constitutively expressed across all tissues may also be examined. Ubiquitin-conjugating enzyme E2 is often used in RT-qPCR (quantitative reverse transcription PCR) and other assays as a constitutive expression control gene (25). A search for gene features that contain BLAST matches to ‘Ubiquitin-conjugating enzyme E2’ (including quotes in the search) yields 123 transcripts from *F. pennsylvanica* as of May 2017. To generate a small exploratory heatmap, we selected 8 transcripts randomly from each set of search results and compared them using the expression visualization heatmap tool (Figure 6D). From the heatmap, we can see that 6 out of 8 heat-shock transcripts have increased expression under several stress conditions, notably heat treatments. The 8 ubiquitin transcripts display more consistent expression across all biomaterials. This example demonstrates the types of data exploration enabled by a combined use of the Tripal Elasticsearch module and the Tripal Analysis Expression module.

## Conclusions

The modular nature of the Tripal software enables new functionality to be easily built and offered as optional add-ons for the Tripal community. The installation of a new module or set of modules can immediately provide the infrastructure for a new genomic content types and provide custom data discovery and visualization interfaces. Here, we described two new extension modules inspired by the need to provide robust transcriptome data access in a Tripal website. First, the Tripal Elasticsearch module services the findable principle of data infrastructure by providing a major advance over prior search solutions. Second, the Tripal Analysis Expression module provides an efficient way for Tripal databases to store and offer public access to gene expression experiment data. This enables exploration of data through visualization of gene expression levels for individual genes and the ability for users to build their own expression heatmaps from their genes of interest, encouraging reuse of existing experimental data. Together, the modules provide significant additional Tripal functionality for users to explore RNASeq data, transcriptome assemblies and transcript expression levels.

## References

[1] Sanderson L.A., Ficklin S.P., Cheng C.H. (2013) Tripal v1.1: a standards-based toolkit for construction of online genetic and genomic databases. Database, 2013, bat075–bat075. 24163125 10.1093/database/bat075 PMC3808541

[2] Ficklin S.P., Sanderson L.-A., Cheng C.-H. (2011) Tripal: a construction toolkit for online genome databases. Database, 2011, bar044. 21959868 10.1093/database/bar044 PMC3263599

[3] Mungall C.J., Emmert D.B. and FlyBase Consortium. (2007) A Chado case study: an ontology-based modular schema for representing genome-associated biological information. Bioinformatics, 23, i337–i346. 17646315 10.1093/bioinformatics/btm189

[4] Lister R., Gregory B.D., Ecker J.R. (2009) Next is now: new technologies for sequencing of genomes, transcriptomes, and beyond. Curr. Opin. Plant Biol., 12, 107–118. 19157957 10.1016/j.pbi.2008.11.004 PMC2723731

[5] Wilhelm B.T., Josette-Renée L. (2009) RNA-Seq—quantitative measurement of expression through massively parallel RNA-sequencing. Methods, 48, 249–257. 19336255 10.1016/j.ymeth.2009.03.016

[6] Todd E.V., Black M.A., Gemmell N.J. (2016) The power and promise of RNA-seq in ecology and evolution. Mol. Ecol., 25, 1224–1241. 26756714 10.1111/mec.13526

[7] Martin L.B.B., Fei Z., Giovannoni J.J. (2013) Catalyzing plant science research with RNA-seq. Front. Plant Sci., 4, 66. 23554602 10.3389/fpls.2013.00066 PMC3612697

[8] Wang Z., Gerstein M., Snyder M. (2009) RNA-Seq: a revolutionary tool for transcriptomics. Nat. Rev. Genet., 10, 57–63. 19015660 10.1038/nrg2484 PMC2949280

[9] Marguerat S., Bähler J. (2009) RNA-seq: from technology to biology. Cell. Mol. Life Sci., 67, 569–579. 19859660 10.1007/s00018-009-0180-6 PMC2809939

[10] Wegrzyn J.L., Main D., Figueroa B. (2012) Uniform standards for genome databases in forest and fruit trees. Tree Genet. Genomes, 8, 549–557.

[11] Wilkinson M.D., Dumontier M., Aalbersberg I.J.J. (2016) The FAIR Guiding Principles for scientific data management and stewardship. Sci. Data, 3, 160018. 26978244 10.1038/sdata.2016.18 PMC4792175

[12] Oshlack A., Robinson M.D., Young M.D. (2010) From RNA-seq reads to differential expression results. Genome Biol., 11, 220. 21176179 10.1186/gb-2010-11-12-220 PMC3046478

[13] Conesa A., Madrigal P., Tarazona S. (2016) A survey of best practices for RNA-seq data analysis. Genome Biol., 17, 13. 26813401 10.1186/s13059-016-0881-8 PMC4728800

[14] Fonseca N.A., Marioni J., Brazma A. (2014) RNA-Seq gene profiling–a systematic empirical comparison. PLoS One, 9, e107026. 25268973 10.1371/journal.pone.0107026 PMC4182317

[15] Vijay N., Poelstra J.W., Künstner A. (2013) Challenges and strategies in transcriptome assembly and differential gene expression quantification. A comprehensive in silico assessment of RNA-seq experiments. Mol. Ecol., 22, 620–634. 22998089 10.1111/mec.12014

[16] Altschul S.F., Madden T.L., Schäffer A.A. (1997) Gapped BLAST and PSI-BLAST: a new generation of protein database search programs. Nucleic Acids Res., 25, 3389–3402. 9254694 10.1093/nar/25.17.3389 PMC146917

[17] Jones P., Binns D., Chang H.Y. (2014) InterProScan 5: genome-scale protein function classification. Bioinformatics, 30, 1236–1240. 24451626 10.1093/bioinformatics/btu031 PMC3998142

[18] Kanehisa M., Goto S. (2000) KEGG: kyoto encyclopedia of genes and genomes. Nucleic Acids Res., 28, 27–30. 10592173 10.1093/nar/28.1.27 PMC102409

[19] Barrett T., Clark K., Gevorgyan R. (2012) BioProject and BioSample databases at NCBI: facilitating capture and organization of metadata. Nucleic Acids Res., 40, D57–D63. 22139929 10.1093/nar/gkr1163 PMC3245069

[20] Avraham S., Tung C.W., Ilic K. (2008) The Plant Ontology Database: a community resource for plant structure and developmental stages controlled vocabulary and annotations. Nucleic Acids Res., 36 (suppl 1), D449–D454. 18194960 10.1093/nar/gkm908 PMC2238838

[21] Lane T., Best T., Zembower N. (2016) The green ash transcriptome and identification of genes responding to abiotic and biotic stresses. BMC Genom., 17, 702. 10.1186/s12864-016-3052-0 PMC5009568 27589953

[22] Ritossa F. (1962) A new puffing pattern induced by temperature shock and DNP in drosophila. Experientia, 18, 571–573.

[23] De Maio A. (1999) Heat shock proteins: facts, thoughts, and dreams. Shock, 11, 1–12. 10.1097/00024382-199901000-00001 9921710

[24] Swindell W.R., Huebner M., Weber A.P. (2007) Transcriptional profiling of Arabidopsis heat shock proteins and transcription factors reveals extensive overlap between heat and non-heat stress response pathways. BMC Genom., 8, 125. 10.1186/1471-2164-8-125 PMC1887538 17519032

[25] Czechowski T., Stitt M., Altmann T. (2005) Genome-wide identification and testing of superior reference genes for transcript normalization in Arabidopsis. Plant Physiol., 139, 5–17. 16166256 10.1104/pp.105.063743 PMC1203353

